# Theranostic Applications of Glycosaminoglycans in Metastatic Renal Cell Carcinoma

**DOI:** 10.3390/cancers15010266

**Published:** 2022-12-30

**Authors:** San Hue Hua, Maximillian Viera, George W. Yip, Boon Huat Bay

**Affiliations:** Department of Anatomy, Yong Loo Lin School of Medicine, National University of Singapore, Singapore 117594, Singapore

**Keywords:** glycosaminoglycans, renal cell carcinoma, metastasis, biomarkers, targeted therapy

## Abstract

**Simple Summary:**

Glycosaminoglycans (GAGs) are a class of carbohydrates that has been closely associated with cancer progression. GAGs have been implicated in cancer cell growth and are known to be involved in cell signaling. As GAGs are present in the extracellular matrix that surround tumor cells (tumor environment), they are intricately involved in the processes of cell migration and invasion, which are crucial for metastasis (spread to distant organs) to take place. Hence, the significance of this review is to explore the potential use of GAGs as biomarkers and therapeutic targets for metastatic renal cancer, which has poor rates of survival and faced with major challenges, that include lack of precise monitoring of disease treatment and effective treatment strategies with minimal toxicity.

**Abstract:**

Renal cell carcinoma (RCC) makes up the majority of kidney cancers, with a poor prognosis for metastatic RCC (mRCC). Challenges faced in the management of mRCC, include a lack of reliable prognostic markers and biomarkers for precise monitoring of disease treatment, together with the potential risk of toxicity associated with more recent therapeutic options. Glycosaminoglycans (GAGs) are a class of carbohydrates that can be categorized into four main subclasses, *viz.*, chondroitin sulfate, hyaluronic acid, heparan sulfate and keratan sulfate. GAGs are known to be closely associated with cancer progression and modulation of metastasis by modification of the tumor microenvironment. Alterations of expression, composition and spatiotemporal distribution of GAGs in the extracellular matrix (ECM), dysregulate ECM functions and drive cancer invasion. In this review, we focus on the clinical utility of GAGs as biomarkers for mRCC (which is important for risk stratification and strategizing effective treatment protocols), as well as potential therapeutic targets that could benefit patients afflicted with advanced RCC. Besides GAG-targeted therapies that holds promise in mRCC, other potential strategies include utilizing GAGs as drug carriers and their mimetics to counter cancer progression, and enhance immunotherapy through binding and transducing signals for immune mediators.

## 1. Metastatic Renal Cell Carcinoma

Renal cell carcinoma (RCC) comprises more than 90% of cases of kidney cancer, with clear cell RCC (ccRCC) being the most common type of RCC and making up the majority of cancer-related deaths [1,2]. The “founding event” of ccRCC is often attributed to a mutation in the von Hippel-Lindau (VHL) tumor suppressor gene [3], although by itself is insufficient to cause ccRCC. The prognosis for RCC is poor, especially for metastatic RCC (mRCC). The overall 5-year survival rate for RCC patients is 74% and decreases to only 8% for patients with mRCC [4,5]. Despite improvements in early detection techniques and considerable progress in systemic treatment, a quarter of patients with localized RCC still develops metastatic deposits at distant sites following surgical removal of the primary tumor (post-nephrectomy) [6,7]. Distant metastases are mostly observed in the lymph nodes, lungs, liver, bone and brain [8].

For early or resectable RCC, nephrectomy is usually performed in the management of this cancer. As RCC is usually resistant to conventional chemotherapy and radiotherapy, the standard treatments for mRCC have been interleukin-2 and interferon cytokine-based therapies, until the availability of targeted therapies [2,9]. Several targeted treatments are available for mRCC management, the most common being tyrosine kinase inhibitors targeting Vascular endothelial growth factor (VEGF) signaling, such as sunitinib [10,11] and sorafenib [12]. Sunitinib is commonly used as a first line treatment option for RCC, with a higher response rate and longer progression-free survival than the conventionally used interferon α as observed during a Phase 3 clinical trial [10]. Moreover, poor-risk RCC patients were also observed to show responses to the drug in the same study. Although sunitinib is still used in VEGFR-targeted therapy for advanced RCC [13], newer generation of multiple VEGF kinase inhibitors, such as Lenvatinib, has been found to be more effective [14].

Another drug target is the mammalian target of rapamycin (mTOR) pathway, which regulates cell proliferation and tumor metabolism [15]. Temsirolimus, a specific inhibitor of mTOR, has been used as both first line and second line treatment options in advanced RCC [16,17]. This intravenous drug has also been shown to achieve prolonged survival over interferon α among mRCC patients in a Phase 3 clinical trial [18,19]. However, despite the clinically beneficial outcomes that these targeted treatments offer, nearly all RCC patients develop resistance to both VEGF-targeted and mTOR-targeted therapies. Thus, a combination of VEGF and mTOR inhibitors has been administered as a strategy to delay drug resistance to either class of the inhibitors [20,21].

Another treatment modality employed is immunotherapy targeting the programmed cell death protein 1 (PD1) and its ligand PDL1, as demonstrated by the effects of the drug nivolumab [22]. PDL1 is overexpressed in cancer cells, and inhibiting PD1-PDL1 interaction promotes T-cell activation and killing of cancer cells. Nivolumab, used as second line treatment for mRCC, has been reported to offer a longer overall survival and higher response rates, with fewer adverse effects and a better quality of life, compared to the mTOR inhibitor Everolimus [23]. A meta-analysis comprising 5121 patients with mRCC from six clinical trials, revealed that Nivolumab plus cabozantinib (an oral inhibitor of multiple tyrosine kinases) was associated with the highest likelihood of patients having maximal overall survival, while the combination of Lenvatinib plus Pembrolizumab (a humanized antibody used in cancer immunotherapy), the highest likelihood of progression free survival [24]. In fact, the European Association of Urology Guidelines has recommended combination therapies of Axitinib plus Pembrolizumab, Cabozantinib plus Nivolumab, and Lenvatinib plus Pembrolizumab for advanced RCC [14]. Another combination therapy which has undergone a phase III clinical trial include, randomization of 873 patients who received either axitinib and avelumab or sunitinib [25]. A recent report has also shown evidence for clinically meaningful and durable benefits in advanced RCC patients treated with Nivolumab plus Ipilimumab (a monoclonal antibody that targets CTLA-4) [26].

A summary of the present treatments available for mRCC is shown Table 1.

Biomarkers for RCC have facilitated identification of patients likely to respond to certain types of treatment and improved prognostic accuracy of cancer metastasis, recurrence, and mortality. As common therapies involve the VEGF pathway, VEGF is a commonly used serum biomarker to predict patient prognosis [27]. However, VEGF expression was observed not to correlate well with receptiveness of the cancer to VEGF inhibitor (Sunitinib) treatment [28]. Serum lactate dehydrogenase (LDH) is involved in the aforementioned mTOR pathway and can potentially be a cheap and convenient biomarker to predict overall survival of RCC patients [29,30]. Urinary markers such as aquaporin 1 and lipid droplet protein perilipin 2 were shown to be elevated in patients with RCC while levels decreased after excision of the tumor [31,32].

## 2. Glycosaminoglycans

**Figure 1:**
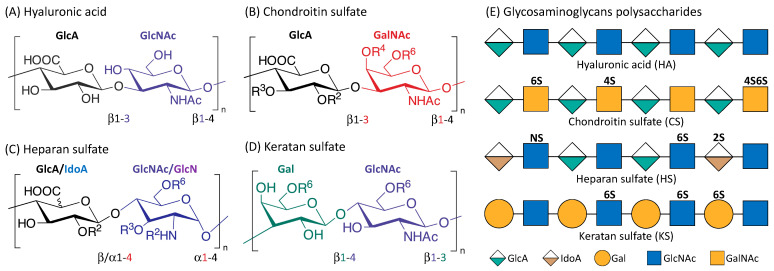
Structure of the four main glycosaminoglycans (GAGs). Disaccharide monomers of Hyaluronic acid (**A**); Chondroitin sulfate (**B**); Heparan sulfate (**C**) and Keratan sulfate (**D**). The possible sulfate sites are denoted with R^i^, the superscript ‘i’ indicate the Carbon position where the sulfate group is esterified; R = H or SO_3_H. Representative Glycosaminoglycans polysaccharides of HA, CS, HS and KS consist of repeating disaccharides monomers with various sulfation patterns (**E**).

Glycosaminoglycans (GAGS) are linear polysaccharides that consist of repeating disaccharide units of uronic acid and an amino sugar [33]. They are found in almost every mammalian tissue, providing structural scaffolding and hydration to the cells [34]. Figure 1 is an illustration of the four main classes of glycosaminoglycans: hyaluronic acid (HA), chondroitin sulfate (CS), heparan sulfate (HS), and keratan sulfate (KS). Monomers of the disaccharide building blocks consist of GlcA (d-glucuronic acid) and GlcNAc (*N*-acetyl-d-glucosamine) for HA; GlcA and GalNAc (*N*-acetyl-d-galactosamine) for CS; GlcA or IdoA (l-iduronic acid) and GlcNAc or GlcN (d-glucosamine) for HS; Gal (d-galactose) and GlcNAc for KS. GAGs are highly polar and negatively charged with the polysaccharide lengths generally varying between 4 and 200 mer [35]. With the exception of HA, GAGs contain sulfate groups attached at specific sites [36]. The sulfate groups are added onto the GAGs chain through post-polymerization modifications [37]. O-sulfotransferases mediate the sulfation of CS and KS while the sulfation of HS is controlled by N-sulfotransferases, C5 epimerases as well as O-sulfotransferases [35,38].

HA (the only GAG known not to have any sulfation sites) has a crucial role in cushioning and lubricating the body that is attributable to its highly hydrophilic property, and is therefore found in abundance in the eyes, joints, and heart valves [39]. HA is also abundant in the skin and important in wound healing [40]. KS is present in the cornea, cartilage, and bones, and associated with disorders such as macular corneal dystrophy and osteoarthritis [41]. HS is usually located in the extracellular matrix (ECM), and is highly involved in tumorigenesis [42,43,44]. CS is an important structural component of cartilage, providing much of their resistance to compression [45]. CS can interact with various biomolecules and form proteoglycans (PGs) with proteins, the major components of the extracellular matrix and drive crucial biological activities. The various sulfation patterns of CS could code for different biological regulatory functions [46,47]. For instance, C4S is known to be important for cartilage regeneration and observed to be downregulated in degraded osteoarthritic cartilage [48]. C4,6S augmented cartilage generation through enhancing type II collagen production [49]. C4,6S, but not C4S or C6S, was reported to be able to interact with several neurotrophic factors to stimulate neurite outgrowth [46].

Interestingly, it is well established that GAGs are involved in cancer cell growth, signalling, and metastasis [34,50]. HA levels are significantly elevated in breast [51], lung [52], and ovarian cancers [53]. Certain sulfation motifs of exogenous CS were shown to induce apoptosis and inhibit the growth of triple negative breast cancer cells [54]. Dermatan sulfate was observed to be elevated with changes in the sulfation profiles in the stroma of certain cancers such as liver [55], lung [56,57], pancreatic [58], colorectal [59] and gastric [60] cancers.

## 3. GAGs and Metastasis

Cancer is a complex disease where malignant cells could acquire the ability to metastasize to distant sites, thus accounting for the majority of cancer-related morbidity and mortality. The fundamental processes of migration and invasion are crucial for cancer metastasis, which is usually the primary cause of death in cancer patients [61]. The ECM, which is composed mainly of GAGs and their PGs, would therefore play a significant role in controlling cell behavior and movement [62]. GAGs are endowed with rigidity property, thus providing structural integrity to the cells and passageways in the ECM between cells [36]. Dysregulation of ECM modelling occurs during tumorigenesis and metastasis, leading to changes in the tumor microenvironment (TME) and loss of tissue homeostasis [63,64]. Disorganization in the ECM GAGs/PGs expression, composition and spatiotemporal distribution are the main causes of the dysregulation of ECM functions and the driver for cancer invasion [65]. GAGs modulate cancer invasion through binding with various growth factors, adhesion molecules and cytokines [66].

**Figure 2:**
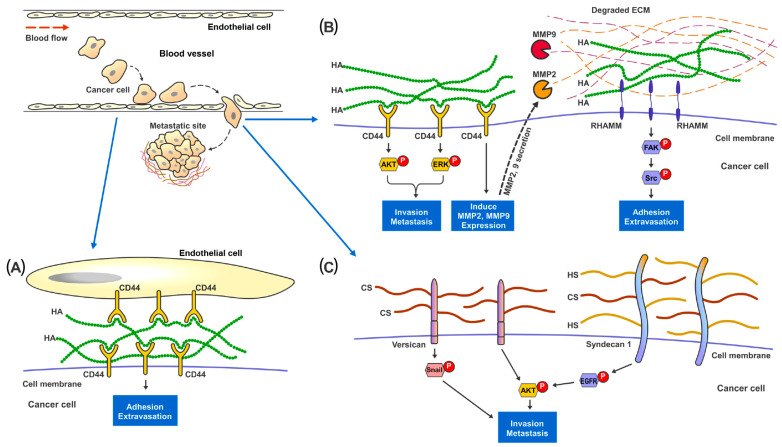
Proposed mechanisms of the involvement of GAGs in cancer metastasis. In metastatic renal cancer, HA, HS and HS present on the cell surface layer promote cancer cell invasion and metastatic capabilities. (**A**) During metastasis, cancer cells traveling in the blood vessel adhere to the endothelial cells through the binding and interaction between cell surface CD44 and HA present in the ECM. (**B**) HA-bound CD44 receptors on cancer cells activates MAPK-ERK1/2 and AKT signaling pathways to promote cell adhesion, migration and invasion. CD44 also increases MMP2 and MMP9 expression and secretion to help digest and remodel ECM proteins at the metastatic site. Cell surface RHAMM, upon the binding of HA in the ECM, activates FAK-Src signaling pathways to help cancer cells migrate through the blood vessel and start colonization at the metastatic site. (**C**) Cell surface proteoglycan of CS and HS, such as Versican and Syndecan-1, promote cancer invasion though activating Snail, EGFR and AKT signaling pathways.

HA is the only GAG that does not form PGs with any protein through covalent bonding, and therefore not sulfated at all [37]. Increased HA synthesis through overexpression of Hyaluronan Synthases (HS) was observed to promote cancer growth and metastasis in xenograft models of breast, prostate, and colon cancers [67,68,69,70]. The presence of HA provides cancer cells with a highly hydrated and malleable ECM which is essential for changes in cell shapes and tissue penetration during invasion [71,72,73]. Pericellular HA surrounding metastatic cancer cells could facilitate adhesion of cancer cells to endothelial cells at the metastatic site [74,75] (Figure 2). Moreover, HA can interact with various cell-surface receptors, notably CD44 and RHAMM, which are well established to be involved in cancer cell survival, motility, and metastasis [76,77]. Studies have shown that disruption of the binding of HA to either CD44 or RHAMM receptors [78,79,80], would suppress the development of metastatic nodules in mice. HA-CD44 interaction has been reported to stimulate Matrix Metalloproteinase 2 (MMP2) and MMP9 expression and their cell-surface presentation [81]. These MMPs play important roles in cancer invasion, as they aid in digesting through the ECM barrier (which is essential in preventing cancer cells from escaping their primary tissue architecture) and in facilitating the growth of cancer cells at metastatic sites [82,83]. HA bound to RHAMM could induce the activation of FAK which is required for actin filament and microtubule rearrangements as well as cancer cell motility [84,85,86,87].

CS, HS and KS can be sulfated and form PGs with the core proteins through covalent binding at the Serine residues [88]. Alterations in cell surface CS expression, sulfation patterns and consequently, ECM-degradative enzymes, such as MMPs, would result in changes of cell invasiveness and disruption of cell-matrix interactions [33,89]. In vitro studies on breast cancer revealed that a higher CS expression in tumor cells was concomitant with increased cell proliferation, migration and invasion [90,91,92,93]. At the tissue level, CS in general was observed to be significantly elevated in the stromal compartment of breast tumors [94,95]. The sulfation patterns of CS also elicited various effects on cancer invasion [89]. For instance, elevated expression of non-sulfated chondroitin in prostate cancer was associated with adverse clinicopathological parameters [96]. C4,6S was noted to suppress cancer cell invasion through inhibiting Wnt/β- Catenin signaling [97] and enhancing the retention of tissue inhibitor of metalloproteinases (TIMP)-3, which disrupt ECM processing and cell mobility [98]. On the other hand, C4,6S expression could promote ovarian cancer metastasis through interacting with VEGF, HGF [99,100] and P-Selectin [101], which support survival of the circulating cancer cells and tissue colonization. C6S bound to CD44 has been reported to promote cancer cell adhesion and migration [102]. C4S is known to suppress cancer invasiveness and inhibit cathepsin S activity which regulate cell–cell and cell-ECM contacts [103,104]. CS proteoglycans (CSPGs) also participate in cancer cells migration, invasion, and metastasis. A large ECM CSPG (Versican) was observed to promote cancer epithelial-to-mesenchymal transition (EMT) and metastasis through EGFR/AKT [105], Snail/PAPSS2 [106] and TGFβ/NK-κB signaling [107] in liver, breast and ovarian cancers. Intracellular CSPG Serglycin has been reported to interact with CD44 [108,109] and activate IL-8 [110] signaling pathways to enhance cell migration and metastasis in lung and breast cancer.

HS is present at the ECM interface to modulate various types of cell-ECM interactions [111]. The capability of HS to bind to various chemokines, growth factors, morphogens, enzymes and ECM proteins, confer functional properties such as controlling cancer migration, EMT and metastasis [34]. Changes in HS sulfation patterns could also affect cancer cell invasion and metastasis [112,113]. Reduction in 6-O-Sulfation of HS has been observed to augment VEGF and FGF induced cell invasion in RCC [114]. On the other hand, an increase in 3-O-Sulfation could enhance the EMT and invasion capacity of pancreatic cancer cells [115]. Cell surface HS/CS Proteoglycans (HSPGs) Syndecan-1 is known to increase cancer stemness and invasiveness through stimulating the Notch and EGFR signaling pathways and regulation of the focal adhesion kinase-Wnt signaling axis [116,117]. In contrast, ECM HSPG Perlecan has been shown to inhibit cancer cell invasion and digested by MMP7 during FAK driven invasion in prostate cancer [118].

KS expression was reported to be increased in pancreatic tumor tissues compared to normal adjacent tissues and stroma, with KS expression being higher in lung metastatic sites compared to the primary pancreatic tumor [119]. KSPG Lumican has been shown to inhibit lung cancer invasion through binding with p120-catenin, which prevent activation of Rho GTPases, FAK and cytoskeletal re-organization [120]. On the other hand, highly glycosylated ECM Lumincan promote colon cancer cells migration through binding with cell surface integrins, and activating actin cytoskeleton remodeling [121,122].

### 3.1. GAGs as Biomarkers in Metastatic RCC

GAGs have been investigated as potential biomarkers in a variety of cancers, examples of which are shown in Table 2.

In ccRCC, GAG biosynthesis is usually dysregulated, leading to changes in secreted GAG profiles (GAG disaccharide composition and sulfation) in the plasma and urine. Comparing ccRCC tissues to their adjacent normal tissues, enzymes involved in the HS biosynthesis were significantly downregulated, while those in the CS biosynthesis pathways were significantly elevated (both at gene expression and protein levels). Likewise, plasma and urine GAG profiles of ccRCC patients were higher in total CS compositions compared to the healthy individuals. Unsulfated CS and 6-O-sulfated HS were also observed to be enriched in metastatic ccRC (mccRCC) plasma and urine samples [133,134]. Moreover, CS and HS, mRCC tumors also had higher cellular hyaluronan expression compared to primary tumor tissues. Cellular hyaluronan has been associated with a higher tumor grade, size, and more advanced stage as well as increased recurrence and mortality rates in RCC patients [135].

To further explore the predictive and prognostic values of GAG in ccRCC, a GAG score based on the free CS and HS concentrations as well as the sulfation compositions of CS and HS in the plasma and urine has been developed. The formula for the score was derived from Gatto et al. in 2018, using a discovery set of 86 samples and a validation set of 160 samples with an area under curve (AUC) of 0.999 and accuracy of 98.9%. The score took into consideration the concentrations of the sulfate, non-sulfate and total CS and HS [136]. The score was able to distinguish between healthy and mccRCC patient samples in clinical studies that were published recently in 2022 and few years back in 2018 [134,136]. It was further observed that the GAG score had high sensitivity and specificity for the occurrence of mccRCC. The GAG score could also be used as an independent prognostic factor for post-operative recurrence of RCC, and identification of patients who had a high or low risk of metastatic recurrence or death in early stage RCC [134,136].

Currently, GAGs and GAG scores are being evaluated in clinical trials around the world as biomarkers for RCC. The Zealand University Hospital in Denmark is in the process of conducting a study for patients with suspected renal tumors, to evaluate the plasma and urine GAG score in order to differentiate GAG scores between RCC patients, oncocytoma patients, and healthy individuals [137]. The Sahlgrenska University Hospital in Sweden is conducting two concurrent studies on the sensitivity and specificity of GAG scores on the early diagnosis of advanced RCC [138] and recurrent RCC, respectively [139]. For recurrent RCC patients, plasma and urine GAGs are being measured and compared against radiological responses for advanced RCC and against post-surgical recurrence, diagnosis, and tumor size. Plasma GAGs were recently ascertained to be highly sensitive diagnostic and prognostic biomarkers in surgically treated RCC, and GAG scores useful for detection of early RCC, prediction and surveillance in recurrent RCC, in a completed clinical trial [136]. A clinical study across multiple locations is also evaluating the use of GAG scores based on serum and urine GAGs for detection of RCC recurrence with comparison to the use of current reference standards [140].

### 3.2. GAGs as Targets for Metastatic RCC

GAGs are known to be usually dysregulated in abundance, sulfation patterns and polysaccharide lengths across various types of cancer [141]. Qazi et al. observed that HA and HS present on the cell surface PG layer (glycocalyx) promoted cancer invasion and metastatic capabilities, in response to the interstitial flow in the tumor ECM present in mRCC [142]. The same investigators demonstrated that digestion of glycocalyx HS and HA using heparinase and hyaluronidase, reduced renal cancer interstitial flow-mediated migration, and inhibited MMP1 and MMP2 expression. Knocking down of *NDST1* (N-sulfotransferase 1), a HS biosynthesis enzyme, suppressed invasion and metastasis of aggressive renal cancer cells in vivo [143]. Additionally, Glypican-1, a cell membrane HS proteoglycan, is the linkage between HS (interstitial flow sensor) and its downstream migration promoting activation of the signaling axis such as the MAPK pathway. A study has shown that knocking down of Glypican-1 would result in reduction of interstitial flow-mediated migration of metastatic renal cancer cells [144]. The above studies suggest that targeting the degradation of glycocalyx, especially extracellular HS/HA is a potential therapeutic strategy to reduce metastasis in aggressive renal cancers. For instance, there is a drug for targeting extracellular HS, naphthalene methanol-D-xyloside (NX), which blocks the assembly of HS side chains onto their PGs [145]. In addition, Pegylated Human recombinant Hyaluronidase (PEGPH20) is a drug used to remove extracellular HA—the main constituent of the tumor ECM physical barrier which limits drugs, monoclonal targeting antibodies (mAb) and immune cell access to the tumor mass [146]. PEGPH20 treatment has been reported to enhance anti-cancer drug efficacy and accessibility of mAb and immune cells to breast and pancreatic tumor sites in both preclinical and clinical settings [146,147,148]. However, despite the potential effects of the glycocalyx targeting drugs and therapies, systemic administration of such compounds would deplete the glycocalyx of endothelial cells and disrupt vascular homeostasis, leading to serious side effects such as vascular leakage, excessive inflammatory responses and sustained nitric oxide induced vasodilation [143,149]. Thus, several targeted strategies are being developed such as nanoparticle delivery to direct the compounds specifically to the tumor mass, so as to reduce the side effects of these therapies [150].

Besides the above-mentioned GAG-targeted therapies in mRCC, there are other promising targeting strategies that has been explored in various types of cancer which could be useful for renal cancers, such as utilizing GAGs as drug or cytokine carriers and their mimetics to interfere with cancer progression [151].

#### 3.2.1. GAGs as Anti-Cancer Drug Carriers

GAGs nanoparticles have been developed to deliver cancer chemotherapeutics compounds into the tumor microenvironment [141,152]. A non-anticoagulant Heparin-deoxycholic acid conjugate was used to deliver doxorubicin with good efficiency and lesser systemic toxicity than free doxorubicin [153]. HA nanoparticles were observed to be effective in delivering drugs to CD44 positive cancer cells [154]. Irinotecan carrying HA is currently under phase II clinical trials for colorectal cancer (NCT01290783) and extensive-stage small cell lung cancer patients [141,155].

#### 3.2.2. GAGs, GAG Derivatives and Mimetics as Anti-Cancer Drugs

Low-molecular-weight heparin (LMWH), usually used for venous thromboembolism prophylaxis during cancer treatment [156], was previously shown to reduce cancer metastasis through binding and blocking of the interactions between cancer cells and several membrane receptors such as Selectins and Integrins [157,158]. Some clinical studies also suggested its benefit to patient survival in advanced lung, colorectal, breast and other solid cancers [159,160]. On the other hand, LMWH treatment has elicited serious side effects such as bleeding and induction of thrombocytopenia due to its anticoagulant characteristic, hindering its long-term usage for cancer treatment, while there are also clinical studies suggesting LMWH having no survival advantage [161,162,163]. Recently, a class of non-anticoagulant HS analogs and Heparinase inhibitors such as Roneparstat, Pixatimod and HS06 are under development with promising anti-cancer effects [164,165,166,167]. Roneparstat is known to reduce tumor growth and angiogenesis through inhibiting Heparinases from digesting Syndecan-1 bound HS and is currently undergoing Phase I clinical trial for advance myeloma [166]. Pixatimod treatment has been shown to reduce cancer growth and metastasis in vivo by inhibiting Heparanase-2 [168] and activating NK and dendritic cells [169,170,171,172]. The drug is currently under Phase II clinical trial for melanoma, lung and colorectal cancers [170]. HS06 has been reported to inhibit cancer-stem-cell self-renewal through activating MAPK signaling [167].

Approaches targeting HA, hyaluronan synthases (HAS) and hyaluronidase are under investigation both preclinically and clinically with promising anti-cancer outcomes. HA small oligosaccharides interfered with HA-CD44 mediated cell survival and invasion signaling pathways and limited tumor growth and metastasis in colon and ovarian cancers [173,174,175]. HA synthesis inhibitors such as 4-methylumbelliferone [176,177], 1,25-dihydroxyvitamin D3 [178] and HAS targeting siRNA [179] have been reported to induce apoptosis and reduce cell growth in lung, liver, breast and colon cancers in vivo. Recombinant human hyaluronidase PH20 (PEGPH20) has been shown to enhance chemotherapy sensitivity by degrading extracellular HA thus improving the accessibility of anti-tumor drugs, mAbs and immune cells [146,180,181,182]. However, a phase III clinical trial of PEGPH20 on metastatic pancreatic cancer did not show any benefit in patient overall and progression-free survival despite the favorable response rates [183].

CS and its derivatives have also been investigated at the preclinical stage for their uses in cancer therapies. Chondroitinase removal of CS was reported to reduce melanoma cell proliferation, invasion, and angiogenesis [184]. Fucosylated CS (FucCS) was observed to inhibit metastasis in animal models though blocking of P- and L-Selectin [185]. Neoglycans-modified CS chains with carbodiimide reduced breast cancer cell growth and induced apoptosis with little toxicity to the normal tissue [186].

#### 3.2.3. GAGs and GAG Derivatives in Cancer Immunotherapy

TME consists of molecules such as TGF-β and its superfamily members, which help the tumor to escape the body’s immune-surveillances, and hinder NK lymphocytes’ access and cytotoxicity activity [187]. GAGs are the main component of the tumor ECM with the capability to bind and transduce signals for immune mediators such as inflammatory chemokines and immune receptors [188,189]. In particular, accumulation of HA in the tumor ECM is known to enhance immune evasion and therefore, a popular target for cancer immunotherapy [190]. HA degradation has been demonstrated to increase PD-L1 antibody uptake, thus, attracting T and NK cells to the tumor in a mouse breast cancer model [191]. Heparanase is a double-edged sword in tumor immunology. The HS-digesting enzyme could sustain chronic pro-tumoral inflammation [192], while Heparanase from the tumor associated macrophages (TAM) are known to promote macrophage infiltration, cytokine secretion and phagocytic activity [193,194]. The Heparanase inhibitor Pixatimod was demonstrated to prevent pro-tumoral macrophage infiltration and improve NK cells activation via dendritic cells [169,195]. A phase I clinical trial has shown that Pixatimod could stimulate the innate immune response leading to increased circulating NK cells in a majority of the patients with advanced solid tumors [196].

**Figure 3:**
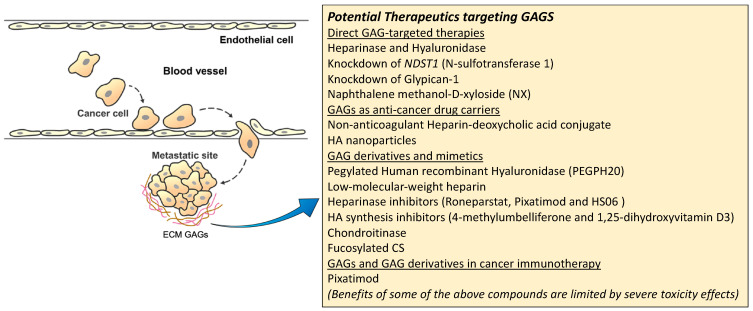
Potential therapeutic targeting GAGs for mRCC.

A summary figure for the potential GAG-targeted therapeutics for mRCC is shown in Figure 3.

## 4. Conclusions

Despite the emergence of new targeted therapies and combinatorial therapy with immuno-oncology agents, which have transformed the therapeutic landscape of advanced RCC [197], there are still challenges faced in the management of mRCC. The availability of reliable prognostic markers for advanced RCC and for precise monitoring of the progress of disease treatment is still an unmet need. Moreover, selection of the optimal treatment option will depend on the outcome to be achieved, as well as identification and validation of predictive biomarkers associated with the desired treatment endpoints [198]. While recent advances in the use of combinatorial therapy to treat mRCC (immunotherapy/immunotherapy or immunotherapy/TKI) have provided opportunities for more efficacious treatments, there is also the added potential risk of toxicity, including protracted and permanent toxicities [199].

GAGS are an important component of the ECM in the tumor microenvironment, which provides crucial biochemical and biomechanical cues mediating cell–cell and cell-matrix interactions, that drives cancer progression and modulate immune responses affecting T cells and other critical elements of the immune system [50]. Interest in understanding the structure-function relationships of GAGs has opened up the fields of glycosaminoglycanomics and heparanomics [200,201]. Recent advances in technologies, including imaging techniques, mass spectrometry, microarrays, and bioinformatics approaches [202,203,204,205], have provided novel biological insights into the glycome and enhanced the field of glycobiology, thereby enabling the knowledge gleaned to be used for better cancer detection and prognostication, and establishing GAG-related cancer therapy [152]. The fact that Muparfostat (PI-88), known to inhibit endo-beta-D-glucuronidase heparanase, has progressed to Phase III clinical trial for hepatocellular cancer, with other efforts also being channeled towards developing small molecule inhibitors and neutralizing antibodies of GAGs as anticancer therapy [141], is ample evidence that GAGS have potential usage in the clinical setting.

Therefore, it is timely to evaluate the utility of GAGs as biomarkers in RCC, especially for risk stratification and strategizing efficacious treatment strategies. Because of the inherent challenges associated with mRCC treatment, GAGS may also serve as exciting alternatives for treating mRCC with the potential of augmenting current immunotherapy and combinatorial therapy protocols. Moreover, of relevance is also the fact that non-clear cell renal cell carcinoma (nccRCC), a highly heterogeneous group of kidney cancers comprising 15 to 30% of renal tumors, has no clearly defined treatment approaches and is under represented in clinical trials (where the focus thus far has been on ccRCC) [206,207]. As developing better therapeutic strategies for each subtype of nccRCC is clearly an urgent need [16,207,208], GAGs would be attractive candidates for further exploration as therapeutic targets for this group of renal cancers, as they participate in fundamental mechanisms that mediate tumor metastasis. Targeting of GAGs could therefore be potentially effective to eradicate both metastatic ccRCC and nccRCC subtypes.

## Figures and Tables

**Table 1:** Current treatments available for mRCC.

Treatment Modality	Selected Therapeutic Options
Cytokine-based therapy	Interleukin-2
	Interferon
TKIs	Sunitinib
	Sorafenib
	Lenvatinib
	Cabozantinib
	Axitinib
mTOR inhibitors	Temsirolimus
	Everolimus
Immunotherapy	Nivolumab
	Pembrolizumab
	Ipilimumab
	Avelumab
Combinatorial therapy	
TKI + Immunotherapy	Axitinib + Pembrolizumab
	Cabozantinib + Nivolumab
	Lenvatinib + Pembrolizumab
	Axitinib + Avelumab
Immunotherapy + Immunotherapy	Ipilimumab + Nivolumab

**Table 2:** GAGS as biomarkers in a variety of cancers.

GAG	Source of GAG	Cancer (Organ of Origin)	Parameters Based on Altered Levels of Specific GAGs and Significance	Reference
HA	Saliva	Head and neck (oral cavity, pharynx and larynx)	Increased salivary HA levels in head and neck squamous cell cancer patients compared to normal	[123]
	Serum	Liver	High serum HA levels associated with shorter recurrence-free survival and overall survival in hepatocellular cancer patients.	[124]
	Urine	Bladder	Increased urinary HA levels in bladder cancer patients (compared with normal), with 83.1% sensitivity, 90.1% specificity and 86.5% accuracy in cancer detection	[125]
	Tissue	Liver	Intrahepatic cholangiocarcinoma is characterized by a significant increase in HA	[126]
HS	Tissue	Skin	High antibody reactivity in cutaneous melanoma tumors compared with nevi	[127]
CS	Tissue	Liver	Raised CS level with a more diverse CS sulfation pattern is associated with poor differentiation status in hepatocellular carcinoma	[126]
	Serum	Ovary	Elevated CS levels in advanced stage and recurrent ovarian epithelial cancer.	[128]
	Tissue	Prostate	Increased CS levels in men with early prostate cancer. Low CS concentration associated with significantly better progression free survival following radical prostatectomy.	[129]
DS	Tissue	Esophagus	CS/DS is significantly increased in esophageal squamous cell carcinoma compared with normal tissue	[130]
KS	Serum	Cartilage	Raised KS levels in chondrosarcoma patients as compared with age- and sex-matched controls.	[131]
	Tissue	Thyroid	Strong labeling of sulfated forms of KS in papillary thyroid cancer but not other types of thyroid neoplasms or in normal tissues.	[132]
